# Allopathic and Naturopathic Medicine and Their Objective Consideration of Congruent Pursuit

**DOI:** 10.1155/2020/7525713

**Published:** 2020-08-06

**Authors:** Ivan Uher, Jaroslaw Cholewa, Marcin Kunicki, Milena Švedová, Iveta Cimboláková, Zuzana Kůchelová, Tatiana Kimáková, Mária Jusková

**Affiliations:** ^1^Institute of Physical Education and Sport, Pavol Jozef Šafárik University, Košice, Slovakia; ^2^Department of Physical Education, J. Kukuczka Academy of Physical Education, Katowice, Poland; ^3^Department of Physical Education, State Higher Vocational School, Raciborz, Poland; ^4^Faculty of Management, Prešov University in Prešov, Prešov, Slovakia; ^5^Department of Public Health and Hygiene, Pavol Jozef Šafárik University, Kosice, Slovakia

## Abstract

In recent years, allopathy (ALP) and naturopathy (NAP) have become a favorite topic, source of argument, and the subject discussed when it comes to choosing treatment modality. Various attempts have been made to elucidate this issue, yet limited advancement has been achieved. To this day, the dispute remains active, and the debate over what to do about it continues to damnify us. The presented qualitative analysis aims to identify existing views or else expand on or uncover already known differences. Ourexamination or position is not about the conflict, finding a superior method (ALP vs. NAP), but aims at inductive reasoning, making broader generalizations from scientific observations. *Subjects and Methods*. We explore the philosophical and psychological foundation of the prevailing ideologies and perspectives in the contemporary society using the Straussian grounded theory approach. The study had no subjects. *Results*. We outline the path for the future direction. *Conclusion*. Our examination concludes that it is essential to acknowledge not only the difference between ALP and NAP but also how they both act on our health. We emphasize that, by identifying our perspective, our inner reflection, and our view on this topic, we can undertake a new paradigm, new road to improve our health, and perhaps the well-being throughout our culture and society.

## 1. Introduction

Men have been looking after their health for thousands of years in innumerable ways, from the early civilizations of Egypt, Asia, and America to medical accomplishments of classical antiquity, the renaissance of medicine in the sixteenth century to the rapid development of the medicine in the nineteenth century, and beyond. Medicine can be categorized into two distinct medical fields: allopathic (or Western, conventional, and orthodox) and naturopathic (Eastern medicine). While ALP and NAP medicine have likely been influencing each other for centuries, they evolved in different parts of the world. Western medicine developed in ancient Greece, while various Eastern practices grew throughout China and India. Whileboth aim for the identical result “optimal health and well-being,” they developed from distinct philosophies that continue to affect, bias, and be practiced today.

ALP medicine forms the basis of many of the world's modern health systems and remains primarily founded on the principles established by the ancient Greeks, being quick, effective, and highly efficient. This is ideal for urgent situations that require demanding and immediate care. Modern medications can rapidly alleviate most negative symptoms and allow people to continue daily life with minimal interruption or discomfort. At times, ALP treats only the symptoms without addressing the root cause of the issue. At the same time, the medications' long-term effect can weigh heavily on the body in the later years of a person's life [1]. ALP focuses on the visible signs and symptoms (infections, pathogens, viruses, bacteria, parasites, etc.). It relies on clinical examination and screening to confirm the diagnosis. Hence, it does not consider, or marginally, the psychological, emotional, aspects, sleeping pattern; therefore, lifestyle of the patient may not be able to relate to any symptoms that are present without any specific cause [1]. ALP divides anatomy into multiple disciplines with respective specialists who treat diseases by focusing on their specialization. However, how that will affect the other body parts, systems, and organs, has not been a point of a large-scale argument. It does not consider, or to a limited extend, how different additional factors interact together [1].

Eastern medicine (NAP) originated mostly throughout Asia; it refers to a range of medical practices employing a wide array of “natural” treatments, including homeopathy, herbalism, acupuncture, and diet and lifestyle counseling. Although these practices have evolved over thousands of years, they still retain many of their original approaches to healing. The two most common forms of Eastern medicine we can find are Chinese medicine and the ancient Indian practice of Ayurveda. NAP believes in going holistic in treating an ailment that considers the physical, mental, emotional, and social well-being (psychosomatic approach) [2], in the reality, so-called multidimensional thinking. Emphasizing disease as a process rather than an entity is defined by principles more than methods or modalities [3].

According to NAP and its proponents [2, 3], sound health can be considered a condition when the body performs at its highest possible level concerning physical, psychological, social, and environmental determinants. Careful consideration is focusing on attitudes, which is a way of thinking and feeling, reflected in our behavior. Hence, the focus is on a person as an integrated human being, with an overall aim of preventing diseases or else curing ailment with their core cause, concentrating on enhancing the effectiveness of the body (capability) systems to facilitate efficient eradication of the illness [4].

Following the above already in the period of Hippocrates was introduced the term “vis medicatrix naturae,” which means healing power, an inherent self-organizing, ordered healing process of living systems which establishes, maintains, and restores health. Simply, it is the power of nature to heal, which we now call alternative or complementary medicine. Furthermore, Dr. Ornish, the pioneer of the modern medicine, advocates that what, how we think, our nutritional habits, how much we move, exercise, sleep, our social connections, the environment we live in, and outmost our lifestyle choices are crucial to our overall health and well-being [5]. Furthermore, after twenty years of practice, Dr. Malerba states that ALP may not tackle the embedded ailment and tendency and hence may not cure individuals entirely. From there creates a possibility that one may experience health issues later and may need more of a substance used for medical treatment, predominately medications, when the underlying problem cause manifests itself again. He also claims that allopathy offers a faster relief but may not completely eradicate the suffering [6].

Notwithstanding the above, many principles and techniques from NAP have become a commonplace in ALP practices, such as treating the “whole person” rather than a symptom or disease or using techniques like meditation as part of the treatment for mental health issues. However, there is a claim that naturopathy, as it is a practice now, does not appear to be science-based nor evidence-based. In summary, we can conclude that ALP has a long secure and prominent place in research and development to discover means and processes that treat diseases effectively with fewer side effects [7, 8]. We can acknowledge that treatment faster results offered by ALP treatment are apparent, which can be favorable in numerous conditions and situations. Furthermore, NAP medicine aims to encourage the body's systems to let it efficiently fight the ills, with the least side effects, taking into consideration the psychosomatic structural and flexible response strategy approach [9, 10].

## 2. Discussion: Line of Reasoning

Our course into ALP and NAP functional health care discussion, argument, will be a view into the core of our perspective. Look that refers to our ideology, belief, thought, attitude, and feeling, supported by the scientific evidence and conviction that the mind can help mobilize the body's healing resources [11, 12]. We cannot understand ALP and NAP issues without realizing the main argument that is being within and around us. What do we mean by a functional health care discussion? A disagreement between professionals, healthcare providers, the general public, etc., each of whom believes that others view than ours (ALP vs. NAP) is incorrect, where mass communication and media tend to amplify this dynamic. We are increasingly concerned with putting a label on others instead of reflecting on our argument. When we are exposed to new views and ideas, we are seldom thinking about whether the concept is right or wrong [13].

Instead, we ask, for example, who has come with the new concept, approach. Such categorization of ideas leads to a means of determining how to think about them. In about reality, our effort to understand ALP and NAP arguments has been replaced by the desire to assign them to narrow categories. Often, one course, “group of thinking,” regards the others as wrong, illegitimate, and even dangerous, wherein the capacity to listen (the active skill) vanishes, and reasonable disagreement collapses. In some spheres of life, for example, technological queries, we are predominantly confronted with openness (open discussion, collective brainstorming, exchange of ideas, etc.) that is establishing a flexible, creative, and productive dialogue—the byproduct of that reality we can observe in an enormous amount of technological innovations.

It appears that, in the health care system, we lost that open-minded, constructive age. However, in some areas of human science, we are a challenge with an open mind, creative thinking, that brings progress, breakthrough, and the opportunity for a new, for instance, David Sinclair, who grapples with some of the most fundamental questions around the science of aging [14], and Csikszentmihalyi who asserts nonconformists provide a vision for our future and the road map on how to get there [15]. Yet, these individuals are not too many among us. For the most part, we find ourselves recycling the same ideas and ideologies around. In comparison to the technological conversation, queries, the healthcare conversation in the contemporary society often leads not only to the exchange of ideas but also to the exchange of doubt, fear, apprehension, and so forth. Such intolerance, lack of openness, receptiveness, and creativity reasonably cannot create an environment for creative problem-solving [16].

Our intention is not to categorize, defend, and mask, instead qualitatively analyze different approaches, understanding, points of view, and the broader philosophy and psychology, which underpin them. We should be aware that a considerable number of people in our society have lost their feeling, sentience, of what is sound health, been immersed in uncertainty and confusion [17, 18]. If we can understand how this confusion arose its motivation and what it is doing to our health, therefore society, we can propel and present an opportunity to move forward [19]. An open discussion, public media network and so forth, can stand as a precursor, pave the way toward “new ideas” that can help make progress, continue to move on in a proper direction [20]. In that contextual reality, we can reset our internal argument and create their discourse anew. However, as a psychological precondition for reconfiguring our way of thinking, we must first adapt to a substantial change in the way we relate emotionally to the way we see, perceive, and think [21], and more specifically, to choose between external adaptation and internal adaptation, the ability to capture and process, or else subjective and objective reality [22, 23]. Goodman, in his works, claims that a person who assimilates his opinions into his identity becomes closed to the critique of those opinions. Any objection to his views is a dubitation and assault on his entity [24]. Following the above, proponents and opponents (ALP vs. NAP) and their contrast of ideas show how they define themselves. Therefore, the position on the conflict is the object of their core identity. Hence, that reality creates an environment least capable of listening to one another [25].

ALP believes that NAP's idea is not just wrong but dangerous, and NAP sees ALP in a similar but opposite fashion. Our opinion is a part of our identity, and another view is a threat to our integrity [24]. That reality prevents us from listening to each other and causes the status quo that consequently excludes, and eliminates an open-minded dialogue. Frequently, we do not view discussions of the conflict as a brainstorming “exercise” to provoke innovation, challenge preconceptions, or nurture new and creative ideas. Instead, an affirmation of identities leads to ideological and intellectual rigidity, which leads to no out result. Even though we know that the presented issue of ALP and NAP medicine is complex, consisting of many different and connecting integrands, the way we think about it is not in any way comprehensive.

Furthermore, as we mentioned, listening as an active skill [25, 26] that comes at a “price” means potentially risking our beliefs. Moreover, we keep a considerable distance between ourselves and our challenging opinions, and that critical distance prevents our views from completely taking over to our identity. Not seldom, we become trapped in our emotions [21]: the feeling of anger, fear, dishonor, and condemnation, these emotions nurture and aggravate, provoking confrontation of the parties involved, where the opposing argument becomes even more robust. On the positive side, this discussion can renew our thinking about the controversy, have the chance to innovate, stop thinking in dichotomies, and start thinking in extent. Giving up the old thought and be prepared for a new [17].

What will happen if we stop seeing health care issues through the ALP and NAP point of view and start viewing them through nondichotomous eyes? Such a change has potentially permitted us to ask entirely new questions. Instead of asking how to end this polemic, we should ask ourselves how to limit it. Perhaps we should start to think in terms of extent. Doing so, we may discover that here, in fact, is already a certain degree of concordance, understanding, and dynamic, and we aim to create more of that. The momentum of that reality has the potential for listening to each other, hence the ensuing collapse of the status quo and resulting open-minded dialogue. However, the argument that makes divisions in the presented issue is by no means grounded on one idea only.

Furthermore, other factors (financial means and at times, personal interests) are also responsible for widening this rift that prevents us from listening to one another. Our way of thinking would be much more productive if we stopped defining the situation as a “problem” and started framing it as an “opportunity” instead [15, 17], because problems are meant to be solved and this problem has no finite solution. An opportunity, however, is not intended for arguing in a heated or angry way but for an acceptance of a new creative path. That approach creates lower and more realistic expectations than the opposition. Even though we will succeed in finding a runaway route, the problem will not disappear; it will only change its form and become an essential part of our reality. This ultimately will not mitigate the existing status quo. In the given context, we must not overlook social responsibility that represents an ethical framework and suggests that an entity, be it an individual or organization, should act for the benefit of the society at large. Social responsibility means sustaining the equilibrium, avoiding, engaging in socially harmful acts, and performing activities that directly advance social goals. Social responsibility must consistently dominate since the competent; qualified actions have consequences on each person and those following us [27]. Healthcare providers should create a sound platform, give strategies, declare a policy, and set the right direction that will serve to all [28].

## 3. Conclusion

Our qualitative analysis aims not to offer a solution to the existing problem but to help instigate discussion, stimulate or give rise to scientific, procreative reasoning, and think about the established topic. We are proposing a practical, nonideological approach as the key to rehabilitating our discourse from its present despondency. Over and above, we know it is not enough merely to promote a pragmatic mode of thinking; it is essential to illustrate that mode as well. Hence, philosophy without practice is by its nature incomplete. We tried to refrain from assigning labels. Based on our qualitative analysis, we attempt to point out the differences between ideologies, pragmatism, and perception of the set topic, outlining how involved sides can make a modification and transition towards objective, more sensitive, realistic picture approach. We can utter that the idea, plan we delineate is not new or original. Its constituting parts are coming from the length and wisdom of life, the continuity of time [29]. What we should do is to merely realize and become fully aware of the reality that manifests itself in our actions.

The presented approaches, views, and solving can be applied to any realm of our life. Our objective is not to persuade but, by analysis, to demonstrate and mostly to encourage to rethink challenges, opinions, ideas, thoughts, and our feelings. In doing so, we cannot hope to end this exchange of diverging or opposite views (can be done on a personal level). Still, we can expect to transform the issue into a different path, vocalize, rationalize the topic and perspective from which we will see, and experience “new-thinking” new dynamics that will benefit all. Going backward or even staying put is not a viable solution to the current scene. The only path forward is the one in which we embrace our potentiality and capability. More in the medical sphere, where we are confronting with daily challenges, one of the significant obstacles to understanding “contemporary medicine” health issues is its complexity and its outstanding wealth of seemingly unrelated details. Where this complexity has led as to specialization, which in turn has further exacerbated the complexity, we should not allow becoming the victims of our rejectionism.

As for now, we favor the notion that although a comprehensive agreement between both ALP and NAP views is unattainable fully yet, partial steps to minimize reverse effects are undoubtedly possible. At the very end, we can conclude that our “lifestyle,” the way we live, is a fundamental integrant of the approach to illness and well-being, followed by ALP and NAP medicine. In treating any health condition, we will undoubtedly have to adjust and change our lifestyle [5]. This may mean changes in our stress level, dietary habits, amount of daily physical activity level, sleeping pattern, and even changes in our living conditions and economic needs. A professional's task is in a joint effort to discover to bring to light the underlying circumstances leading to our condition, where the way we see (we see what we are preparing to see) underlines our decision outcome [29–32]. Where there is a “new” reinvigorating outlook, perspective is always needed to see something “fundamentally” new.

## References

[1] Demetriou M. C., Demetriou A. (2007). *Integrating Complementary and Conventional Medicine*.

[2] Block D. M. (2003). *The Revolution of Naturopathic Medicine: Remaining True of Our Philosophy*.

[3] Hechtman L. (2011). *Clinical Naturopathic Medicine*.

[4] World Naturopathic Federation Report. http://www.worldnaturopathicfederation.org, 2015, Appendix II. Naturopathic History

[5] Ornish D., Ornish O. (2019). *Undo It. How Simple Lifestyle Changes Can Reverse Most Chronic Diseases*.

[6] Malerba L. (2010). *Green Medicine: Challenging the Assumptions of Conventional Health Care*.

[7] Peters D. (2007). *New Medicine*.

[8] Collier J. A. B. (2017). *Oxford Handbook of Clinical Medicine*.

[9] Hahnemann S. (1996). *Organon of the Medical Art*.

[10] Lloyd I. (2009). *The History of Naturopathic Medicine a Canadian Perspective*.

[11] Cousins N. (1989). *Head First, the Biology of Hope*.

[12] Uher I. (2011). Plurality of methods and approaches that constitute concept of Well-being. *Czech Kinanthropology*.

[13] Isaksen S. G., Dorval K. B., Treffinger D. J. (2011). Creative approaches to problem solving. *A Framework for Innovation and Change*.

[14] Sinclair D. (2019). *Lifespan*.

[15] Csikszentmihalyi M. (1996). *Creativity: Flow and the Psychology of Discovery and Invention*.

[16] Fogler S., LeBlanc S. E., Rizzo B. (2014). *Creative Problem Solving*.

[17] Tolle E. (1997). *The Power of Now*.

[18] Uher I., Švedová M. (2013). Long and prosperous life paradigm. *Journal of Physical Activity Review*.

[19] Malagodi E. F., Jackson K. (1989). Behavior analysts and cultural analysis: troubles and issues. *The Behavior Analyst*.

[20] Ferrer R., Klein W., Lerner J., Reyna V., Keltner D. (2014). Emotions and health decision-maing: extending the appraisal tendency framework to improve health and healthcare. *Behavioral Economics and Public Health*.

[21] Goleman D. (2011). *Emotional Intelligence: Translation*.

[22] Pivovarník J. (2018). *Dual Concentration in Karate and Its Possible Use in Sport Diagnostics*.

[23] Csikszentmihalyi M. (1990). *Flow: The Psychology of Optimal Experience*.

[24] Goodman M. (2018). *Catch-67*.

[25] Allen M. B. (1982). *Listening the Forgotten Skill*.

[26] Uher I., Švedová M. (2012). Listening and perception of others. *Journal of Law and Economics and Organization*.

[27] Crowther D., Aras G. (2008). *Corporate Social Responsibility*.

[28] Antiel R. M., James K. M., Egginton J. S., Sheeler R. D. (2014). Specialty, political affiliation, and perceived social responsibility are associated with U.S. Physical Reactions to Health Care Reform Legislation. *Journal of General Internal Medicine*.

[29] Křivohlavý J. (2009). *Psychology of Health (Psychologie Zdraví)*.

[30] Chopra D. (1991). *Perfect Health: The Complete Mind/Body Guide*.

[31] Chopra D. (1987). *Creating Health, beyond Prevention, toward Perfection*.

[32] Nakonečný M. (1996). *Motivation of Human Behavior (Motivace Lidského Chování)*.

